# Comparative Analysis of Buccal and Lingual Bone Thicknesses Following Immediate and Delayed Loading of Posterior Mandibular Implants

**DOI:** 10.7759/cureus.117200

**Published:** 2026-09-30

**Authors:** Garima S Dev, Abhishek Balani, Vinay Kharsan, Abhishek Karan, Swati Sahu

**Affiliations:** 1 Department of Oral and Maxillofacial Surgery, New Horizon Dental College and Research Institute, Bilaspur, IND

**Keywords:** alveolar bone loss, cone-beam computed tomography, dental implants, mandible, osseointegration

## Abstract

Introduction: Peri-implant bone preservation is a critical determinant of long-term implant survival. Although both immediate and delayed loading protocols have demonstrated favorable clinical outcomes, their influence on longitudinal buccal and lingual bone remodeling remains an area of investigation. This study compared peri-implant bone thickness changes following immediate and delayed loading in posterior mandibular implants using cone-beam computed tomography (CBCT).

Methods: A prospective study was conducted on 80 patients who received single posterior mandibular implants. The patients were divided into two groups: immediate loading (n = 40) and delayed loading (n = 40). Buccal bone thickness (BBT) and lingual bone thickness (LBT) were measured at the crestal, middle, and apical levels on standardized CBCT scans obtained immediately after implant placement and before prosthetic loading (T1), at six months (T2), and at 12 months (T3) after implant placement. Two-way mixed repeated-measures analysis of variance (RM-ANOVA), independent-samples t-test, and multiple linear regression analyses were performed, with statistical significance set at p < 0.05.

Results: The baseline demographic characteristics were comparable between the groups. The immediate loading group demonstrated significantly greater insertion torque (43.60 ± 4.83 vs. 33.29 ± 6.05 Ncm, p < 0.001) and implant stability quotient (72.17 ± 4.46 vs. 69.38 ± 4.21, p = 0.005). BBT decreased significantly in both groups during follow-up, with greater crestal bone loss in the immediate loading group (0.57 ± 0.17 mm) than in the delayed loading group (0.27 ± 0.09 mm). LBT also decreased over time but exhibited a smaller magnitude of remodeling than the buccal cortical plate. Two-way mixed RM-ANOVA demonstrated significant group × time interactions for both BBT and LBT (p < 0.001). Multiple linear regression identified immediate loading as the strongest independent predictor of 12-month crestal buccal bone loss (β = 0.660, p < 0.001), whereas increasing age showed a modest protective association (β = -0.212, p = 0.008).

Conclusions: Both loading protocols demonstrated successful peri-implant bone adaptation during the first postoperative year. However, immediate loading was associated with greater peri-implant bone remodeling, particularly in the crestal region, whereas delayed loading resulted in better preservation of BBT and LBT. Careful patient selection and adequate primary implant stability are essential when immediate loading protocols are employed.

## Introduction

Dental implants have become the preferred treatment modality for replacing missing teeth because of their high survival rates, predictable functional outcomes, preservation of alveolar bone, and positive influence on patient acceptance and treatment willingness, making them a widely accepted option for oral rehabilitation [1]. However, long-term implant success depends not only on successful osseointegration but also on the maintenance of healthy peri-implant hard and soft tissues [2]. Marginal bone stability is a critical determinant of implant longevity, esthetic outcomes, and resistance to peri-implant diseases. Among peri-implant structures, the buccal and lingual cortical plates undergo continuous remodeling following implant placement, and excessive bone resorption may compromise implant stability and long-term prognosis [3,4].

The loading protocol is considered one of the key factors influencing peri-implant bone remodeling. Immediate loading, in which the implant is restored within 48-72 hours after placement, offers several clinical advantages, including reduced treatment time, improved patient comfort, and early restoration of function and esthetics [5]. Conversely, delayed loading allows for a conventional healing period before prosthetic rehabilitation, theoretically minimizing micromotion during osseointegration and potentially preserving the peri-implant bone [6]. Although both protocols have demonstrated high implant survival rates, the influence of loading time on buccal bone thickness (BBT) and lingual bone thickness (LBT) remains controversial, with previous studies reporting inconsistent findings owing to differences in study design, implant systems, loading criteria, and radiographic assessment methods [7].

Cone-beam computed tomography (CBCT) enables an accurate three-dimensional evaluation of the peri-implant bone and allows standardized measurements of buccal and lingual alveolar bone thickness at different implant levels [8]. Longitudinal CBCT assessment provides valuable insights into the pattern of peri-implant bone remodeling over time and facilitates objective comparisons between different loading protocols [9]. However, limited evidence is available regarding the changes in BBT and LBT at multiple vertical levels in posterior mandibular implants following immediate and delayed loading.

This study aimed to compare the longitudinal changes in buccal peri-implant alveolar bone thickness (BBT) and lingual peri-implant alveolar bone thickness (LBT) following immediate and delayed loading protocols in posterior mandibular implants using CBCT. The objectives were to evaluate changes in BBT and LBT at the crestal, middle, and apical levels over a 12-month follow-up period; compare the magnitude of peri-implant bone remodeling between the immediate- and delayed-loading groups; assess the interaction between loading protocol and time on peri-implant bone changes; and determine the influence of demographic and implant-related factors, including age, smoking status, implant dimensions, insertion torque, and implant stability quotient (ISQ), on 12-month crestal peri-implant bone loss using multivariable regression analysis. We hypothesized that delayed loading would be associated with greater preservation of peri-implant bone thickness, particularly at the crestal level, because the additional healing period before prosthetic loading may allow greater bone maturation and adaptation before exposure to occlusal forces.

## Materials and methods

Study design, setting, location, duration, and ethical approval

This prospective observational cohort study was conducted at the Department of Oral and Maxillofacial Surgery, New Horizon Dental College and Research Institute, Bilaspur, India, between July 2024 and March 2026. Patients were allocated to the immediate or delayed loading groups solely on the basis of the prosthetic loading protocol already recorded in their clinical treatment plans; no randomization or investigator-directed intervention was performed. The prosthetic loading protocol was determined by the treating clinician as part of the preexisting clinical treatment plan and was not assigned by the investigators. Primary implant stability was assessed clinically using insertion torque and ISQ. In routine clinical decision-making, implants achieving insertion torque ≥35 Ncm and ISQ ≥70 were considered suitable for immediate restoration, whereas implants not meeting these stability criteria underwent conventional healing before delayed loading. The study therefore prospectively observed patients according to their clinically determined loading protocol without investigator-directed modification of treatment. The study was designed and reported in accordance with the Strengthening the Reporting of Observational Studies in Epidemiology guidelines [10]. Ethical approval was obtained from the Institutional Ethics Committee (NHDCRI/2024/MDS/OSMF/15-ECC, dated 04-06-2024) before the commencement of the study, and written informed consent was obtained from all participants before their enrollment.

Sample size calculation and sampling technique

The required sample size was estimated using G*Power software (version 3.1.9.7, Heinrich Heine University Düsseldorf, Düsseldorf, Germany). An a priori power analysis was performed for an independent-samples comparison, assuming an effect size of 0.65 (Cohen’s d), an alpha error of 0.05, and statistical power of 85%. Because no prior study had quantified longitudinal changes in buccal and lingual peri-implant bone thickness under the same conditions, the effect size was not derived from empirical data; instead, it was selected as a planning assumption based on conventional benchmarks for a medium-to-large effect and the magnitude of difference considered clinically meaningful. The calculation indicated a minimum of 74 patient/implant pairs. To allow for potential incomplete records or exclusions, 80 patient/implant pairs (40 per group) were enrolled. Consecutive nonprobability sampling was employed, whereby all eligible patients presenting to the Department of Oral and Maxillofacial Surgery and scheduled for single posterior mandibular implant placement during the study period were considered for inclusion. Each patient contributed one implant; therefore, the patient/implant pair was the unit of analysis.

Study population and eligibility criteria

Patients presenting to the department and scheduled to undergo single implant placement in the mandibular premolar or molar region were screened for eligibility. Patients who met the inclusion criteria were prospectively enrolled after providing written informed consent. Clinical and radiographic evaluations, including CBCT, were performed at baseline (T1, immediately after implant prosthesis loading), six months post-loading (T2), and 12 months postloading (T3) according to the study protocol.

Eligible participants were adults aged 20-70 years with adequate bone volume to receive standard-diameter implants (3.75-4.50 mm) without the need for bone augmentation. Only patients who received a single implant-supported restoration in the posterior mandible and demonstrated adequate primary implant stability, with documented insertion torque and ISQ measurements at the time of surgery, were included. Participants were required to maintain satisfactory oral hygiene and comply with all scheduled follow-up visits during the 12-month observation period.

Patients were excluded if implant placement was performed in fresh extraction sockets or grafted sites, if multiple adjacent implants were planned, or if they had uncontrolled systemic diseases affecting bone metabolism, previous radiotherapy involving the head and neck region, current or previous bisphosphonate therapy, pregnancy, active periodontal or peri-implant infection, or any condition likely to compromise osseointegration. Patients with poor-quality CBCT scans caused by motion artifacts or metallic scatter, or those who failed to complete the scheduled follow-up examinations, were also excluded from the final analysis. Implants were placed in healed edentulous sites rather than immediately following tooth extraction; patients requiring implant placement in fresh extraction sockets were excluded from the study.

Group allocation

Following eligibility screening, patients were classified into two groups according to the prosthetic loading protocol documented in the original treatment records. The immediate-loading group comprised 40 patients whose implants received screw-retained provisional or definitive restorations within 48-72 hours after implant placement, with the restorations adjusted to provide light occlusal contact. The delayed-loading group comprised 40 patients who underwent conventional implant healing for approximately three to four months before prosthetic loading.

Implant placement procedure

All implant surgeries were performed by an experienced oral and maxillofacial surgeon (GSD) using a standardized surgical protocol under local anesthesia. Following crestal incision and flap elevation or flapless surgery, depending on the clinical situation, osteotomy preparation was completed using sequential drills under copious sterile saline irrigation according to the manufacturer’s recommended drilling sequence. All patients received Touareg-S dental implants (ADIN Dental Implant Systems Ltd., Afula, Israel), with implant diameters ranging from 3.75 to 4.50 mm and lengths between 8 and 12 mm. Implant placement was performed using a calibrated surgical motor with controlled insertion torque. Primary implant stability was assessed immediately after implant placement by recording insertion torque (Ncm) using the torque-control function of the implant motor and measuring the ISQ using resonance frequency analysis with the Osstell IDx (Osstell AB, Gothenburg, Sweden). The prosthetic loading protocol was determined by the treating surgeon as part of the clinical treatment plan and was not assigned by the investigators. In routine clinical decision-making, implants achieving an insertion torque of ≥35 Ncm and an ISQ of ≥70 were considered suitable for immediate restoration and received screw-retained provisional restorations within 48-72 hours of implant placement with light occlusal contact. Implants not meeting these stability criteria underwent conventional healing and received healing abutments or cover screws before prosthetic restoration after approximately three to four months. All patients received identical postoperative instructions, analgesics, antimicrobial therapy when indicated, and routine maintenance visits according to the departmental protocol.

CBCT image acquisition

Radiographic evaluation was performed using a NewTom VGi Evo CBCT system (QR S.r.l., Verona, Italy). All CBCT scans were acquired using identical exposure parameters throughout the study period to ensure measurement standardization. The imaging protocol used an 8 × 8 cm field of view, a tube voltage of 110 kVp, a tube current of 5 mA, an isotropic voxel size of 0.20 mm, and an exposure time of approximately 15 seconds. During image acquisition, the patients were positioned with the Frankfort horizontal plane parallel to the floor and the midsagittal plane perpendicular to the floor, according to the manufacturer's recommendations. Digital Imaging and Communications in Medicine datasets were exported and reconstructed using standardized image settings before quantitative analysis.

Radiographic measurements

Linear measurements were performed using NNT Software (version 10, QR S.r.l., Verona, Italy). Before measurement, each CBCT volume was reoriented such that the long axis of the implant was perfectly aligned in the sagittal and coronal planes. Cross-sectional images passing through the center of the implant were generated, and all measurements were performed perpendicular to the long axis of the implant. BBT and LBT were measured at three standardized vertical reference levels corresponding to the implant platform (crestal level), 3 mm apical to the implant platform (middle level), and 6 mm apical to the implant platform (apical level) (Figure 1).

**Figure 1:**
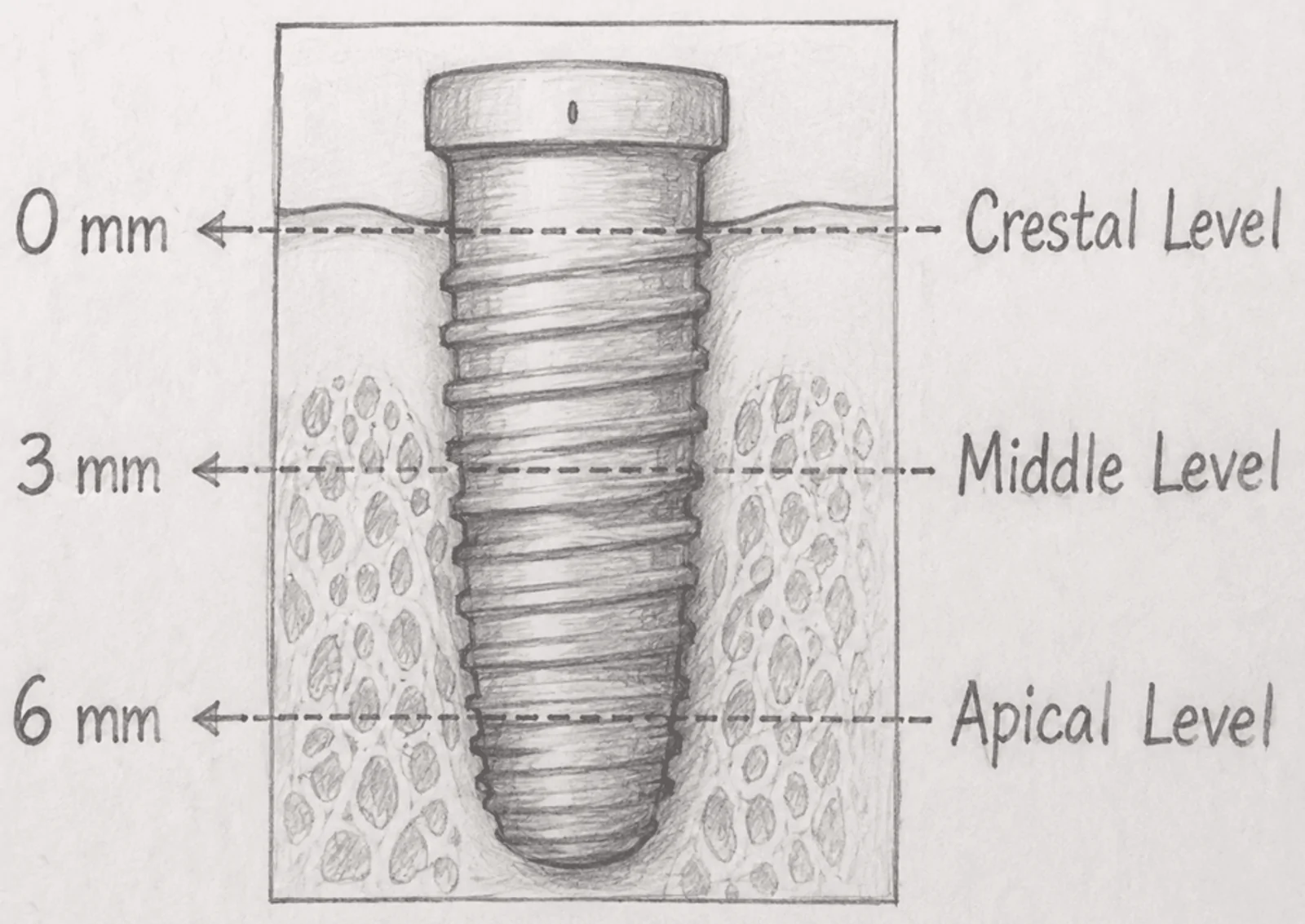
Schematic of the standardized reference levels used for bone thickness measurements: 0 mm (crestal level), 3 mm (middle level), and 6 mm (apical level) relative to the implant platform Image credit: This figure was created by the authors using Canva (Canva Pty Ltd, Sydney, Australia)

BBT was defined as the shortest horizontal distance from the outer implant surface to the external border of the buccal cortical plate, whereas LBT was measured from the implant surface to the external border of the lingual cortical plate at the corresponding level (Figure 2).

**Figure 2:**
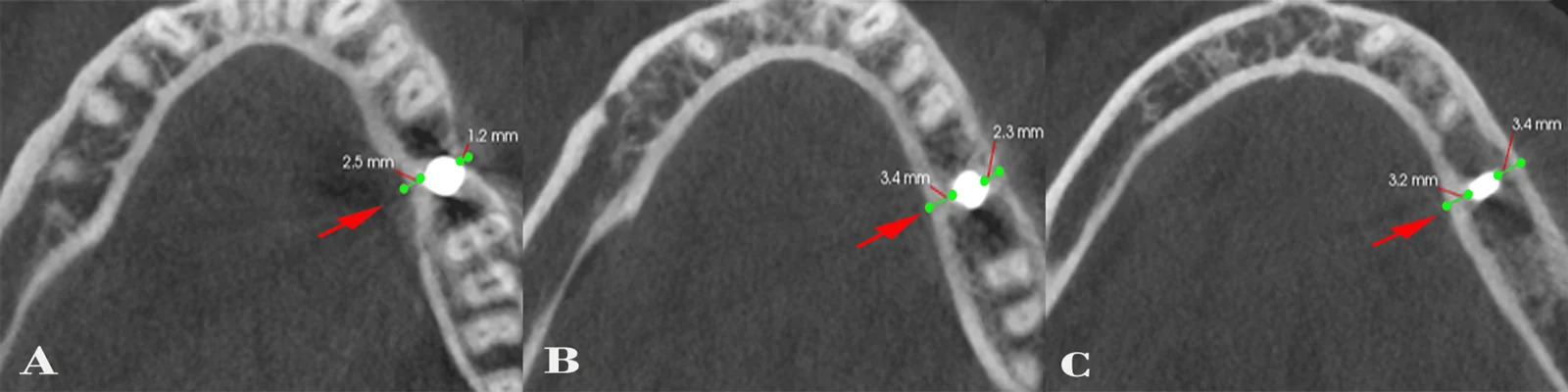
Cone-beam computed tomography images showing measurements of buccal and lingual alveolar bone thicknesses around a mandibular implant. (A) Axial view showing buccal and lingual alveolar bone thicknesses at the implant platform (0 mm; crestal level). (B) Axial view showing buccal and lingual alveolar bone thicknesses at 3 mm apical to the implant platform (middle level). (C) Axial view showing buccal and lingual alveolar bone thicknesses at 6 mm apical to the implant platform (apical level) Red arrows indicate the lingual surface of the alveolar bone Representative cone-beam computed tomography (CBCT) cross-sectional images were obtained from the patient in the study cohort.

Measurements were recorded to the nearest 0.01 mm at baseline (T1), six months (T2), and 12 months (T3) post-loading. Longitudinal changes in peri-implant bone thickness were calculated by subtracting the follow-up measurements from the baseline values.

Data collection

Demographic, clinical, surgical, and radiographic data were prospectively collected during the study period. The demographic variables included patient age, sex, and smoking status. The clinical and implant-related variables included implant diameter, implant length, insertion torque, ISQ, loading protocol, and follow-up duration. The radiographic variables included BBT and LBT measured at the crestal, middle, and apical levels on CBCT images obtained at T1, T2, and T3. All data were verified for completeness and accuracy before being entered into a standardized electronic database for the statistical analysis.

Examiner calibration and reliability

Radiographic measurements were independently performed by two calibrated examiners (AB, VK) who were blinded to the loading protocol throughout the measurement process. Before the commencement of the study, both examiners underwent calibration using ten CBCT scans that were not included in the final analysis. To determine intraexaminer reliability, 20 randomly selected CBCT scans were reevaluated after a two-week interval. Interexaminer reliability was determined by comparing measurements independently obtained by both examiners. We assessed measurement reliability using the intraclass correlation coefficient (ICC). The analysis demonstrated excellent reproducibility for all the radiographic measurements. The intraexaminer ICC was 0.94 (95% confidence interval (CI): 0.90-0.97), and the inter-examiner ICC was 0.91 (95% CI: 0.87-0.95), indicating excellent agreement and high measurement consistency. Whenever discrepancies greater than 0.30 mm were observed, the final measurement was established following a consensus between both examiners.

Primary and secondary outcomes

The primary outcome of the study was the change in crestal BBT from baseline (immediately after implant placement) to 12 months, measured in millimeters on standardized CBCT cross-sections. Secondary outcomes included the changes in BBT at the middle and apical levels, the changes in LBT at the crestal, middle, and apical levels over the same 12-month period, the longitudinal within-group changes in buccal and LBT across the three evaluation time points (baseline, six months, and 12 months), the interaction effects between loading protocol and time on peri-implant bone thickness, and the independent predictors of 12-month crestal buccal bone loss identified through multiple linear regression analysis.

Confounders

The following potential confounders were considered and adjusted for in the multiple linear regression analysis of 12-month crestal buccal bone loss: patient age, smoking status, baseline crestal BBT, insertion torque, and loading protocol. Other factors such as implant diameter and length were compared at baseline between the groups. Variables that could not be fully controlled (for example, occlusal scheme, bone density, implant surface characteristics, and prosthetic design) were acknowledged as potential residual confounders in the study limitations.

Statistical analysis

Statistical analyses were performed using IBM Statistical Package for the Social Sciences Statistics for Windows (version 26.0, IBM Corp., Armonk, NY). The normality of continuous variables was assessed using the Shapiro-Wilk test. Continuous variables are presented as mean ± standard deviation, whereas categorical variables are presented as frequencies and percentages. We compared baseline demographic and clinical characteristics between the immediate- and delayed-loading groups using independent-samples t-tests for continuous variables and chi-square tests for categorical variables. Intergroup differences in cumulative changes in BBT and LBT were evaluated using independent-samples t-tests. Longitudinal changes across T1, T2, and T3 were evaluated using repeated-measures analysis of variance (RM-ANOVA), with Bonferroni-adjusted post hoc comparisons for significant time effects.

The interaction between loading protocol and time was assessed using a two-way mixed RM-ANOVA, with loading protocol specified as the between-subject factor and time as the within-subject factor. Mauchly’s test was used to assess sphericity, and the Greenhouse-Geisser correction was applied when the assumption of sphericity was violated. Multiple linear regression was performed with 12-month crestal buccal bone loss as the dependent variable. The prespecified covariates were age, smoking status, baseline crestal BBT, insertion torque, and loading protocol. Insertion torque was retained as the quantitative measure of primary implant stability, whereas ISQ was not simultaneously included because both variables represent related aspects of primary implant stability, and ISQ was also part of the clinical criteria used for selection of the immediate-loading protocol. Implant diameter was not included in the prespecified regression model because it was considered an implant-design characteristic rather than a primary covariate in the clinical model. The potential for overlap among loading protocol, insertion torque, and ISQ was considered during model specification; however, because ISQ was not included in the final model, no formal multicollinearity assessment involving ISQ was performed. Statistical significance was set at a two-tailed p-value of <0.05 for all analyses.

## Results

Eighty patients were included in the analysis, with 40 (50.0%) assigned to the immediate loading group and 40 (50.0%) to the delayed loading group. Baseline demographic characteristics were comparable between groups (p > 0.05); however, implants in the immediate loading group exhibited significantly greater insertion torque (43.60 ± 4.83 vs. 33.29 ± 6.05 Ncm, p < 0.001) and a higher ISQ (72.17 ± 4.46 vs. 69.38 ± 4.21, p = 0.005) than those in the delayed loading group (Table 1).

**Table 1:** Baseline demographic and clinical characteristics of the study population Data are presented as mean ± standard deviation or n (%) An independent samples t-test was used for continuous variables, and a chi-square test was used for categorical variables ^*^p < 0.05 was considered statistically significant ISQ: implant stability quotient; df: degrees of freedom; SD: standard deviation

Variable	Group A, immediate (n = 40)	Group B, delayed (n = 40)	Test statistic (df)	p value
Age (years), mean ± SD	43.07 ± 9.71	46.61 ± 9.13	t (78) = 1.68	0.096
Sex, male, n (%)	24 (60.0%)	19 (47.5%)	χ² (1) = 0.80	0.370
Smoking status, n (%)	8 (20.0%)	12 (30.0%)	χ² (1) = 0.60	0.439
Implant length (mm), mean ± SD	10.41 ± 1.25	9.87 ± 1.30	t (78) = 1.89	0.062
Implant diameter (mm), mean ± SD	4.16 ± 0.27	4.01 ± 0.26	t (78) = 2.37	0.020^*^
Insertion torque (Ncm), mean ± SD	43.60 ± 4.83	33.29 ± 6.05	t (78) = 8.43	<0.001^*^
ISQ at implant placement, mean ± SD	72.17 ± 4.46	69.38 ± 4.21	t (78) = 2.88	0.005^*^

Mean BBT declined progressively from T1 to T3 at all three vertical levels in both groups. In the immediate loading group, crestal BBT decreased from 1.52 ± 0.31 to 0.95 ± 0.33 mm, with smaller reductions at the middle (1.81-1.53 mm) and apical (2.18-2.08 mm) levels. The delayed loading group showed a similar but less pronounced pattern (crestal: 1.56-1.29 mm), with resorption again greatest at the crestal level and minimal at the apical level in both groups. LBT also decreased over the 12-month follow-up in both groups, though the magnitude of change was consistently smaller than that observed for the buccal plate. In the immediate group, crestal LBT declined from 2.08 ± 0.28 to 1.78 ± 0.30 mm, while the delayed group showed a smaller reduction (2.00-1.87 mm). Middle- and apical-level LBT remained comparatively stable across both groups, with the delayed loading group showing only marginal change at the apical level (2.58-2.54 mm), as given in Table 2.

**Table 2:** Thickness of alveolar bone at different reference level across multiple time points in study groups Data values are presented as mean ± standard deviation only T1: baseline at implant prosthesis loading; T2: six months postloading; T3: 12 months post-loading; BBT: buccal bone thickness; LBT: lingual bone thickness; crestal: implant platform level; middle: 3 mm apical to platform; apical: 6 mm apical to platform; SD: standard deviation

Level	Time points	Group A, immediate (n = 40)		Group B, delayed (n = 40)	
		BBT, mean ± SD	LBT, mean ± SD	BBT, mean ± SD	LBT, mean ± SD
Crestal	T1	1.52 ± 0.31	2.08 ± 0.28	1.56 ± 0.29	2.00 ± 0.32
	T2	1.16 ± 0.32	1.93 ± 0.29	1.40 ± 0.29	1.92 ± 0.33
	T3	0.95 ± 0.33	1.78 ± 0.30	1.29 ± 0.30	1.87 ± 0.32
Middle	T1	1.81 ± 0.28	2.41 ± 0.24	1.87 ± 0.29	2.33 ± 0.29
	T2	1.65 ± 0.29	2.32 ± 0.24	1.79 ± 0.30	2.29 ± 0.29
	T3	1.53 ± 0.28	2.26 ± 0.29	1.74 ± 0.31	2.28 ± 0.26
Apical	T1	2.18 ± 0.23	2.53 ± 0.26	2.03 ± 0.26	2.58 ± 0.23
	T2	2.13 ± 0.23	2.50 ± 0.27	2.00 ± 0.26	2.58 ± 0.23
	T3	2.08 ± 0.24	2.47 ± 0.27	1.97 ± 0.26	2.54 ± 0.22

Comparison of longitudinal bone changes demonstrated significantly greater peri-implant remodeling in the immediate loading group than that in the delayed loading group. Crestal buccal bone loss was 0.57 ± 0.17 mm in the immediate loading group and 0.27 ± 0.09 mm in the delayed loading group (p < 0.001). Similarly, crestal lingual bone loss was 0.30 ± 0.11 and 0.13 ± 0.08 mm, respectively (p < 0.001). Although statistically significant differences were also observed at the middle and apical levels, the magnitude of bone loss progressively decreased toward the implant apex (Table 3).

**Table 3:** Intergroup comparison of changes in buccal and lingual bone thickness from baseline to 12 months Data are presented as mean ± standard deviation An independent-samples t-test was used; Cohen's d = effect size ^*^p < 0.05 was considered statistically significant Δ: change from T1 to T3; df: degree of freedom; BBT: buccal bone thickness; LBT: lingual bone thickness

Parameter	Level	Group A, Δ mean ± SD (mm)	Group B, Δ mean ± SD (mm)	t (df = 78)	p value	Cohen's d
ΔBBT	Crestal	0.57 ± 0.17	0.27 ± 0.09	9.82	<0.001^*^	2.20
ΔBBT	Middle	0.28 ± 0.08	0.13 ± 0.06	9.47	<0.001^*^	2.12
ΔBBT	Apical	0.10 ± 0.06	0.06 ± 0.04	3.23	0.002^*^	0.72
ΔLBT	Crestal	0.29 ± 0.09	0.12 ± 0.06	9.67	<0.001^*^	2.16
ΔLBT	Middle	0.13 ± 0.06	0.07 ± 0.05	5.20	<0.001^*^	1.16
ΔLBT	Apical	0.06 ± 0.06	0.03 ± 0.04	2.59	0.011^*^	0.58

Mixed RM-ANOVA demonstrated significant effects of time for all buccal and lingual measurements (p < 0.001), together with significant group × time interactions, indicating different patterns of peri-implant bone remodeling between the loading protocols. The strongest interaction was observed for crestal BBT (partial η² = 0.408, p < 0.001) (Table 4).

**Table 4:** Two-way mixed repeated measures ANOVA for the effects of loading protocol and time on bone thickness Data are presented as F values and partial η². Greenhouse-Geisser correction was applied when the assumption of sphericity was violated ^*^p < 0.05 was considered statistically significant df: degrees of freedom; BBT: buccal bone thickness; LBT: lingual bone thickness; ANOVA: analysis of variance

Outcome/level	Source	F value	df	p value	Partial η²
BBT					
Crestal	Group	9.71	1, 78	0.003^*^	0.111
	Time	421.86	2, 156	<0.001^*^	0.844
	Group × time	53.74	2, 156	<0.001^*^	0.408
Middle	Group	4.82	1, 78	0.031^*^	0.058
	Time	361.61	2, 156	<0.001^*^	0.823
	Group × time	48.39	2, 156	<0.001^*^	0.383
Apical	Group	6.11	1, 78	0.016^*^	0.073
	Time	85.53	2, 156	<0.001^*^	0.523
	Group × time	5.33	2, 156	0.006^*^	0.064
LBT					
Crestal	Group	0.00	1, 78	0.972	<0.001
	Time	303.51	2, 156	<0.001^*^	0.796
	Group × time	52.03	2, 156	<0.001^*^	0.400
Middle	Group	0.55	1, 78	0.461	0.007
	Time	125.19	2, 156	<0.001^*^	0.616
	Group × time	14.61	2, 156	<0.001^*^	0.158
Apical	Group	1.45	1, 78	0.232	0.018
	Time	37.61	2, 156	<0.001^*^	0.325
	Group × time	4.95	2, 156	0.008^*^	0.060

Bonferroni-corrected paired comparisons confirmed that crestal bone loss was significant across all time intervals in the immediate loading group (all p < 0.05), including the T2-T3 interval, which reflects continued resorption after the early healing phase. In the delayed group, crestal changes remained largely significant, but middle- and apical-level comparisons frequently failed to reach significance after correction, particularly the T2-T3 interval. This pattern indicates that clinically meaningful, statistically robust bone loss was concentrated at the crestal level and within the immediate loading protocol (Table 5).

**Table 5:** Post hoc pairwise comparisons of alveolar bone thickness across time points (Bonferroni-corrected paired t-tests) ^*^p < 0.05 was considered statistically significant BBT: buccal bone thickness; LBT: lingual bone thickness; df: degree of freedom; T1: baseline at implant prosthesis loading; T2: six months postloading; T3: 12 months postloading; Cohen d: partial eta squared

Outcome/level	Group	Comparison	t (df = 39)	p value (Bonferroni)	Cohen's dz
BBT					
Crestal	A (Immediate)	T1 vs. T2	8.22	<0.001^*^	1.30
		T1 vs. T3	9.39	<0.001^*^	1.48
		T2 vs. T3	5.18	<0.001^*^	0.82
	B (Delayed)	T1 vs. T2	5.16	<0.001^*^	0.82
		T1 vs. T3	5.86	<0.001^*^	0.93
		T2 vs. T3	2.90	0.018^*^	0.46
Middle	A (Immediate)	T1 vs. T2	5.30	<0.001^*^	0.84
		T1 vs. T3	7.48	<0.001^*^	1.18
		T2 vs. T3	4.20	<0.001^*^	0.66
	B (Delayed)	T1 vs. T2	2.78	0.025^*^	0.44
		T1 vs. T3	3.40	0.005^*^	0.54
		T2 vs. T3	2.24	0.093	0.35
Apical	A (Immediate)	T1 vs. T2	2.02	0.151	0.32
		T1 vs. T3	3.34	0.006^*^	0.53
		T2 vs. T3	2.23	0.095	0.35
	B (Delayed)	T1 vs. T2	1.03	0.926	0.16
		T1 vs. T3	1.57	0.374	0.25
		T2 vs. T3	1.02	0.945	0.16
LBT					
Crestal	A (Immediate)	T1 vs. T2	5.15	<0.001^*^	0.81
		T1 vs. T3	7.76	<0.001^*^	1.23
		T2 vs. T3	5.03	<0.001^*^	0.80
	B (Delayed)	T1 vs. T2	2.28	0.085	0.36
		T1 vs. T3	2.93	0.017^*^	0.46
		T2 vs. T3	1.66	0.312	0.26
Middle	A (Immediate)	T1 vs. T2	3.24	0.007^*^	0.51
		T1 vs. T3	3.36	0.005^*^	0.53
		T2 vs. T3	1.64	0.326	0.26
	B (Delayed)	T1 vs. T2	1.41	0.500	0.22
		T1 vs. T3	1.68	0.304	0.27
		T2 vs. T3	1.03	0.924	0.16
Apical	A (Immediate)	T1 vs. T2	0.89	1.000	0.14
		T1 vs. T3	1.68	0.305	0.27
		T2 vs. T3	1.51	0.414	0.24
	B (Delayed)	T1 vs. T2	0.00	1.000	0.00
		T1 vs. T3	1.13	0.799	0.18
		T2 vs. T3	1.71	0.288	0.27

Multiple linear regression analysis showed that immediate loading was the strongest independent predictor of 12-month crestal buccal bone loss (β = 0.660, p < 0.001), whereas increasing age was associated with reduced bone loss (β = -0.212, p = 0.008). Baseline bone thickness, insertion torque, and smoking status were not significant predictors after multivariable adjustment (Table 6).

**Table 6:** Multiple linear regression analysis for predictors of 12-month crestal buccal bone loss Model summary: R² = 0.612, adjusted R² = 0.586, F (5, 74) = 23.37, p < 0.001 Multiple linear regression analysis was performed using the enter method ^*^p < 0.05 was considered statistically significant BBT: buccal bone thickness; CI: confidence interval; R²: coefficient of determination; adjusted R²: adjusted coefficient of determination; F: overall model statistic

Predictor	B (unstandardized)	Standard error	β (standardized)	t value	p value	95% CI
Constant	0.346	0.167	-	2.08	0.041^*^	0.014 to 0.678
Age (years)	-0.0045	0.002	-0.212	-2.73	0.008^*^	-0.008 to -0.001
Baseline BBT crestal, T1 (mm)	0.034	0.052	0.050	0.65	0.520	-0.070 to 0.137
Insertion torque (Ncm)	0.002	0.003	0.083	0.81	0.420	-0.003 to 0.008
Smoking status (ref: yes = 1)	0.022	0.036	0.047	0.61	0.547	-0.050 to 0.093
Loading protocol (ref: Immediate = 1)	0.263	0.041	0.660	6.41	< 0.001^*^	0.181 to 0.345

## Discussion

The present study evaluated longitudinal changes in peri-implant BBT and LBT following immediate and delayed prosthetic loading protocols in posterior mandibular implants using standardized CBCT analysis. The principal finding was that both loading groups demonstrated progressive peri-implant bone remodeling during the first year after implant placement, with greater reductions in bone thickness observed in the immediate-loading group, particularly at the crestal region. The apical region remained comparatively stable in both groups. The greater crestal remodeling observed with immediate loading represents an association within the present observational cohort and should be interpreted in the context of the nonrandomized treatment allocation and the higher baseline insertion torque and ISQ in the immediate-loading group. Although loading protocol remained significantly associated with 12-month crestal BBT after adjustment for selected covariates, these findings should not be interpreted as evidence of a causal effect of loading protocol on peri-implant bone remodeling.

Successful osseointegration depends on establishing a stable bone-to-implant interface that can adapt to functional forces. After implant placement, physiological bone remodeling occurs as part of healing, involving initial woven bone formation followed by replacement with mature lamellar bone [11]. During this period, mechanical loading may influence the balance between bone formation and resorption. Immediate loading exposes the implant-bone interface to occlusal forces during the early healing phase, which may alter the mechanical environment of the surrounding cortical bone [12]. Although controlled mechanical stimulation may contribute to bone adaptation, excessive stress concentration, particularly around the crestal cortical bone, has been proposed as a potential factor influencing peri-implant remodeling [13]. In the present study, the immediate-loading group demonstrated greater crestal bone thickness reduction than the delayed-loading group. However, given the observational design and the higher baseline insertion torque and ISQ in the immediate-loading group, this difference should be interpreted as an association rather than as evidence that immediate loading directly caused greater bone remodeling. The observed pattern may reflect the combined influence of loading protocol, primary implant stability, and other biological and clinical factors that were not completely controlled in the present study.

Covani et al. [14] reported that both immediate and delayed implant placement were associated with successful healing of peri-implant defects, although buccolingual crestal bone remodeling occurred in both groups. Their findings suggested that the timing of implant placement may influence peri-implant bone remodeling; however, because their study compared implant placement protocols rather than prosthetic loading protocols, these findings are not directly comparable with the present study and should not be interpreted as evidence for an effect of immediate vs. delayed loading. An important observation in the present study was that buccal bone demonstrated greater remodeling than the lingual cortical plate, irrespective of the loading protocol. This finding is consistent with previous observations that the buccal peri-implant bone may undergo substantial dimensional remodeling following implant placement [15,16]. Benic et al. [16], for example, evaluated buccal bone dimensions around immediately placed implants using CBCT over a seven-year follow-up and reported persistent long-term remodeling of the buccal bone. These findings provide contextual evidence for the susceptibility of the buccal peri-implant region to dimensional changes, although differences in implant placement timing, follow-up duration, and study design preclude direct comparison with the present loading-related findings.

The predominance of crestal bone remodeling observed in the present study is consistent with the biomechanical characteristics of implant-supported restorations. Occlusal forces are concentrated primarily at the crestal portion of the implant, where the implant first engages the cortical bone. This region experiences the highest stress concentration, particularly during lateral and oblique loading, resulting in greater remodeling than the middle and apical portions of the implant [17,18]. As the applied forces dissipate along the implant body, the mechanical stimulus decreases toward the apex, explaining the comparatively limited bone changes observed at greater depths. These findings further support the concept that crestal bone preservation remains the primary determinant of long-term implant success [19].

The significant interaction between loading protocol and time indicates that peri-implant bone remodeling is a dynamic biological process rather than a single postoperative event [19]. Bone adaptation continues during the first year following implant placement, although the pattern and magnitude of remodeling may differ between loading protocols [6]. Although delayed loading has traditionally been considered to provide an uninterrupted healing period before prosthetic loading, evidence from randomized clinical trials indicates that different loading protocols can produce comparable implant survival and marginal bone outcomes when appropriate case selection and adequate primary stability are achieved [20]. In the present study, however, the delayed-loading group demonstrated greater preservation of peri-implant bone thickness during follow-up than the immediate-loading group. This finding indicates an association between loading protocol and the longitudinal pattern of peri-implant bone remodeling in the present cohort, but it should be interpreted in the context of the nonrandomized treatment allocation and the higher baseline insertion torque and ISQ observed in the immediate-loading group. Thus, the observed difference cannot be attributed to the loading protocol alone.

Another notable finding was that the loading protocol remained the strongest independent predictor of crestal bone remodeling after adjustment for demographic and clinical variables, whereas insertion torque, smoking status, and baseline bone thickness were not independently associated with bone loss. These findings suggest that mechanical loading exerts a greater influence on early peri-implant bone adaptation than several commonly investigated patient- or implant-related factors [21]. Interestingly, younger age was associated with greater bone remodeling, which may reflect higher physiological bone turnover and more active remodeling processes rather than compromised healing processes. This observation highlights that greater radiographic remodeling should not necessarily be interpreted as a negative biological response but rather as part of the adaptive process that occurs during functional loading.

The findings of the present study are broadly consistent with those of previous clinical investigations, demonstrating high survival rates and predictable outcomes for both immediate and delayed loading protocols. While systematic reviews have generally reported no clinically important differences in implant survival or marginal bone loss between loading strategies, the present study observed greater peri-implant crestal bone remodeling in the immediate loading group, suggesting that the loading protocol may influence early peri-implant bone adaptation under specific clinical conditions [14,20]. Earlier studies have similarly shown that peri-implant bone changes are predominantly confined to the coronal portion of the implant and that adequate primary stability remains essential for predictable and immediate loading [21,22]. Collectively, these observations support the growing consensus that immediate loading is a reliable treatment option when appropriate case selection, sufficient insertion torque, controlled occlusion, and meticulous surgical techniques are achieved.

From a clinical perspective, our findings emphasize the importance of careful patient selection when considering immediate loading. Achieving high primary implant stability, minimizing excessive occlusal loading during the healing period, and preserving the crestal cortical plate through atraumatic surgical techniques may reduce peri-implant bone remodeling. Regular radiographic follow-up using standardized CBCT protocols can facilitate the early detection of peri-implant bone changes and enable timely intervention before clinically significant bone loss occurs.

The present study had certain limitations. First, the study was conducted at a single institution and had a one-year follow-up period, which may limit the generalizability of the findings and the assessment of long-term peri-implant bone remodeling and implant survival. Second, treatment allocation was nonrandomized, and immediate loading was selected partly based on predefined primary stability criteria, resulting in differences in baseline insertion torque and ISQ between the loading groups. Therefore, the greater reduction in crestal BBT observed with immediate loading may partly reflect selection-related confounding rather than the loading protocol alone. Although multivariable regression was performed to adjust for selected potential confounders, residual confounding cannot be completely excluded. In addition, the potential influences of occlusal scheme, bone density, implant surface characteristics, prosthetic design, and patient-related behavioral factors could not be completely controlled. Future prospective multicenter studies with larger sample sizes, standardized allocation or appropriate matching strategies, and longer observation periods are required to further elucidate the longitudinal association between loading protocols and peri-implant bone remodeling and implant survival.

## Conclusions

In this prospective observational study, immediate loading was associated with greater longitudinal reductions in buccal and lingual peri-implant bone thickness, particularly at the crestal level, than delayed loading. However, because treatment allocation was nonrandomized and differed in baseline implant stability, these findings should not be interpreted as evidence of a causal effect of loading protocol. Although multivariable regression adjusted for selected baseline and clinical factors, residual confounding cannot be excluded. Further randomized or carefully controlled prospective studies are warranted to clarify the independent effect of loading protocol on peri-implant bone remodeling.
